# The suitability of biotypes Q and B of *Bemisia tabaci* (Gennadius) (Hemiptera: Aleyrodidae) at different nymphal instars as hosts for *Encarsia formosa* Gahan (Hymenoptera: Aphelinidae)

**DOI:** 10.7717/peerj.1863

**Published:** 2016-04-18

**Authors:** Xin Liu, Youjun Zhang, Wen Xie, Qingjun Wu, Shaoli Wang

**Affiliations:** Department of Plant Protection, Institute of Vegetables and Flowers, Chinese Academy of Agricultural Sciences (CAAS), Beijing, China

**Keywords:** *Encarsia formosa*, Host instar, Suitability, *Bemisia tabaci* biotype B, *Bemisia tabaci* biotype Q, Biological control

## Abstract

*Encarsia formosa* Gahan (Hymenoptera: Aphelinidae) is a solitary endoparasitoid that is commercially reared and released for augmentative biological control of whiteflies infesting greenhouse crops. In most areas in China, the invasive and destructive whitefly *Bemisia tabaci* (Gennadius) (Hemiptera: Aleyrodidae) biotype Q has replaced *B. tabaci* biotype B and has become dominant between the two. A better understanding of the suitability of different nymphal instars of *B. tabaci* biotypes Q and B as hosts for *E. formosa* is needed to improve the use of this parasitoid for biological control. Parasitism of the four nymphal instars of *B. tabaci* biotypes Q and B by the commercial strain of *E. formosa* mass reared on *Trialeurodes vaporariorum* (Westwood) (Hemiptera: Aleyrodidae) was assessed in the laboratory. The results indicated that *E. formosa* parasitized and successfully developed on all instars of both biotypes but performed best on the 3rd instar of *B. tabaci* biotype B and on the 2nd, 3rd, and 4th instars of *B. tabaci* biotype Q. The host-feeding rate of the adult parasitoid was generally higher on nymphal instars of *B. tabaci* biotype Q than on the corresponding nymphal instars of biotype B and was significantly higher on the 2nd and 3rd instars. For both whitefly biotypes, the parasitoid’s immature developmental period was the longest on the 1st instar, intermediate on the 2nd and 3rd instars, and the shortest on the 4th instar. The parasitoid emergence rate was significantly lower on the 1st instar than on the other three instars and did not significantly differ between *B. tabaci* biotype B and biotype Q. Offspring longevity was greater on the 3rd and 4th instars than on the 1st instar and did not significantly differ between the two *B. tabaci* biotypes. The results indicate that commercially-produced *E. formosa* can parasitize all instars of *B. tabaci* biotypes B and Q, making this parasitoid a promising tool for the management of the two biotypes of *B. tabaci* present in China.

## Introduction

The sweetpotato whitefly, *Bemisia tabaci* (Gennadius) (Hemiptera: Aleyrodidae), is a serious pest of many important vegetable, field, and ornamental crops in the tropics and subtropics. *Bemisia tabaci* not only directly damages plants by feeding and by secreting honeydew (which supports sooty mould), but also damages plants by transmitting more than 100 begomoviruses that greatly reduce crop yields (De Barro et al., 2011). Recent studies suggest that *B. tabaci* is a species complex containing more than 30 morphologically indistinguishable cryptic species (De Barro et al., 2011; Boykin et al., 2013; Lee et al., 2013). The two most invasive and destructive species are the Middle East-Asia Minor I species and the Mediterranean species, formerly referred to as biotype B and Q, respectively (De Barro et al., 2011; Pan et al., 2012). Here, we refer to them as biotypes. Biotype B was first recorded in China in the early 1990s, and its subsequent spread resulted in severe crop damage and losses throughout China (Luo et al., 2002). Biotype Q was first detected in Yunnan, China in 2003 (Chu et al., 2006). Studies indicate that biotype Q has quickly replaced biotype B and has become the dominate *B. tabaci* biotype in many regions of China (Chu et al., 2010; Teng, Wan & Chu, 2010; Pan et al., 2011). Biotype Q is currently considered a more serious pest than biotype B because of its greater virus transmission efficiency (Pan et al., 2012; Liu et al., 2013) and higher insecticide resistance (Rauch & Nauen, 2003; Luo et al., 2010; Xie et al., 2014).

Biological control is an attractive alternative for the sustainable management of *B. tabaci* populations. *Encarsia formosa* Gahan (Hymenoptera: Aphelinidae) is a uniparental (thelytokous) hymenopteran parasitoid (Agekyan, 1982) that has been used commercially for the augmentative biological control of whiteflies infesting greenhouse crops, including *B. tabaci* and the greenhouse whitefly *Trialeurodes vaporariorum* (Westwood) (Hemiptera: Aleyrodidae) (Hoddle, Van Driesche & Sanderson, 1998; Gerling, Alomar & Arno, 2001; Grille et al., 2012; Liu, Stansly & Gerling, 2015). Although studies have demonstrated that *E. formosa* can effectively parasitize whiteflies (Hu, Gelman & Blackburn, 2002; Zhang et al., 2003; Takahashi, Filho & Lourencao, 2008; Zang & Liu, 2008; Wang et al., 2015), some factors including the parasitoid strain (Zhang et al., 2003), the host instar (Zhang et al., 2003; Urbaneja & Stansly, 2004; Wang et al., 2015), and the species on which the parasitoid was reared (Johanna, Rott & Silvia, 2007; Dai et al., 2014; Wang et al., 2015) might substantially affect the fitness of *E. formosa*.

Another concern regarding the fitness of *E. formosa* and its use for augmentative biological control of *B. tabaci* is that many studies of this host-parasitoid interaction have used *E. formosa* reared on biotypes B or Q (Zhang et al., 2003; Dai et al., 2014; Liu et al., 2014), despite the fact that commercial *E. formosa* is mass reared on *T. vaporariorum* (Natskova, 1987; Cheng et al., 1989). Some reports suggest that the progeny of *E. formosa* distinguish its hosts after preimaginal conditioning. Dai et al. (2014) found that *E. formosa* parasitized more whitefly nymphs if it attacked the same host species on which it had been reared. Also, the host-feeding and oviposition potential of *E. formosa* reared on *T. vaporariorum* were significantly higher on the castor whitefly, *Trialeurodes ricini* (Misra) (Hemiptera: Aleyrodidae), than on *B. tabaci* B biotype (Wang et al., 2015). Therefore, some doubts arise about the efficacy of *E. formosa* mass reared on *T. vaporariorum* for controlling *B. tabaci* biotype B or Q.

The main objective of this study was to evaluate the suitability of different nymphal instars of *B. tabaci* biotypes B and Q as hosts for *E. formosa* mass reared on *T. vaporariorum*. We used laboratory assays to evaluate the following parameters concerning *E. formosa* fitness: parasitism rate; host-feeding rate; the immature developmental period; emergence rate; and longevity of progeny.

## Materials and Methods

### Whitefly and parasitoid cultures

The biotype B population used in this study was originally collected from an infested cabbage (*Brassica oleracea*) field in Beijing, China in 2004, and the biotype Q (Q1 genotype) population was originally collected from poinsettia (*Euphorbia pulcherrima*) plants in Beijing, China in 2009. Biotypes were confirmed by random amplified polymorphic DNA polymerase chain reaction (RAPD-PCR) (Luo et al., 2002; Rao et al., 2011) and the genotype of biotype Q was identified as Q1 using the PCR-RFLP analysis of the *COI* (*mitochondrial cytochrome oxidase I*) amplicons (Parrella, Scassillo & Giorgini, 2012). Both biotype populations were maintained on tomato plants in a greenhouse at 26 ± 1 °C with 70 ± 5% relative humidity (RH) and 16 h of photophase. The greenhouse was located at the Institute of Vegetables and Flowers, Chinese Academy of Agricultural Sciences, Beijing, China.

The strain of *Encarsia formosa* used in this study was obtained from the Beneficial Insects Research Center, Shangdong Academy of Agricultural Sciences. The parasitoid had been maintained on a population of *T. vaporariorum* reared on tobacco plants for more than 10 years.

### Infested experimental plants

Potted tomato plants (*Lycopersicon esculentum* Mill, variety “No. 9 Zhongza”) with 6 to 8 fully expanded leaves were used as host plants for biotypes B and Q. These plants were grown in a greenhouse under the same conditions described for the whiteflies. Twenty newly emerged adults (10 females and 10 males) of *B. tabaci* biotype B or Q were placed on the abaxial surface of one leaflet of a tomato plant. The 20 whitefly adults were contained in a leaf clip-cage (2.5 cm diameter, one clip cage per plant). After 24 h, the adults were removed, and all plants containing whiteflies eggs were maintained in a climate chamber at 27 ± 1°C with 70 ± 5% RH and 14 h of photophase. The eggs were examined daily with a stereoscopic microscope (SZ2-ILST, OLYMPUS) until they developed into 1st, 2nd, 3rd, or 4th instar nymphs. The number of nymphs was then adjusted to 30 per clip cage for the experiment. This required approximately 160 tomato plants, each with one clip cage.

### Parasitism and adult host-feeding rates on different instars and biotypes of *Bemisia tabaci*

Caged tomato leaflets infested with 30 nymphs (biotype Q or B at 1st, 2nd, 3rd, or 4th instar) were obtained as described in the previous section. Subsequently, one newly emerged *E. formosa* female (24 h old) was introduced into each clip-cage and then removed after 24 h. The percentages of nymphs that were parasitized or fed by *E. formosa* adults were determined at 8–10 d after the *E. formosa* females were removed from the clip-cages. Nymphs were recorded as parasitized when the parasitoid brown pupae were visible inside the body of the nymphs and the nymphs were recorded as fed by *E. formosa* adults if their bodies were desiccated and flat under stereoscopic microscope. Each combination of biotype and nymphal instar was represented by approximately 20 replicate cages.

### Immature developmental period, emergence rate, and longevity of *Encarsia formosa* on different instars and biotypes of *Bemisia tabaci*

Leaves in clip-cages with parasitoid pupae were obtained as described in the previous sections and were then detached from the plants. The leaf petioles were wrapped in cotton wool, which was then saturated with distilled water; the petioles and cotton wool were placed in plastic Petri dishes (diameter 8.5 cm, height 1.5 cm, one leaf per dish). A filter paper disk saturated with distilled water was also placed beneath each leaf. The pupae in the Petri dishes were examined daily with a stereoscopic microscope until adult parasitoids emerged. Immature developmental period (time from oviposition through adult emergence) and the number of parasitoids emerged were recorded. Upon emergence, the parasitoid adults were individually collected and maintained in 1-mL plastic tubes without food supply; these parasitoid adults were checked daily until they died, at which time their longevity was recorded.

### Data analysis

Data were first checked for normality and were transformed when necessary to meet the assumption of normal distribution. Proportional data including parasitism rate of *E. formosa*, host-feeding rate of *E. formosa* adults, and emergence rate of *E. formosa* were arcsine-square root transformed before analyses. Two-way ANOVAs were used to assess the effects of *B. tabaci* biotype and instar on the following parameters: parasitism rate of *E. formosa*, host-feeding rate of *E. formosa* adults, immature developmental period of *E. formosa*, emergence rate of *E. formosa* adults from pupae, and longevity of *E. formosa* adults. Results were plotted with Sigmaplot version 12.0. Tukey’s test was used to separate treatment means when a main effect or interaction was significant. Statistical analyses were performed with SPSS (version 19.0, Chicago, IL, USA) (Jiao et al., 2013; Liu et al., 2014; Zhang et al., 2016).

## Results

### Parasitism rate of *Encarsia formosa* as affected by *Bemisia tabaci* biotype and instar

**Figure 1:**
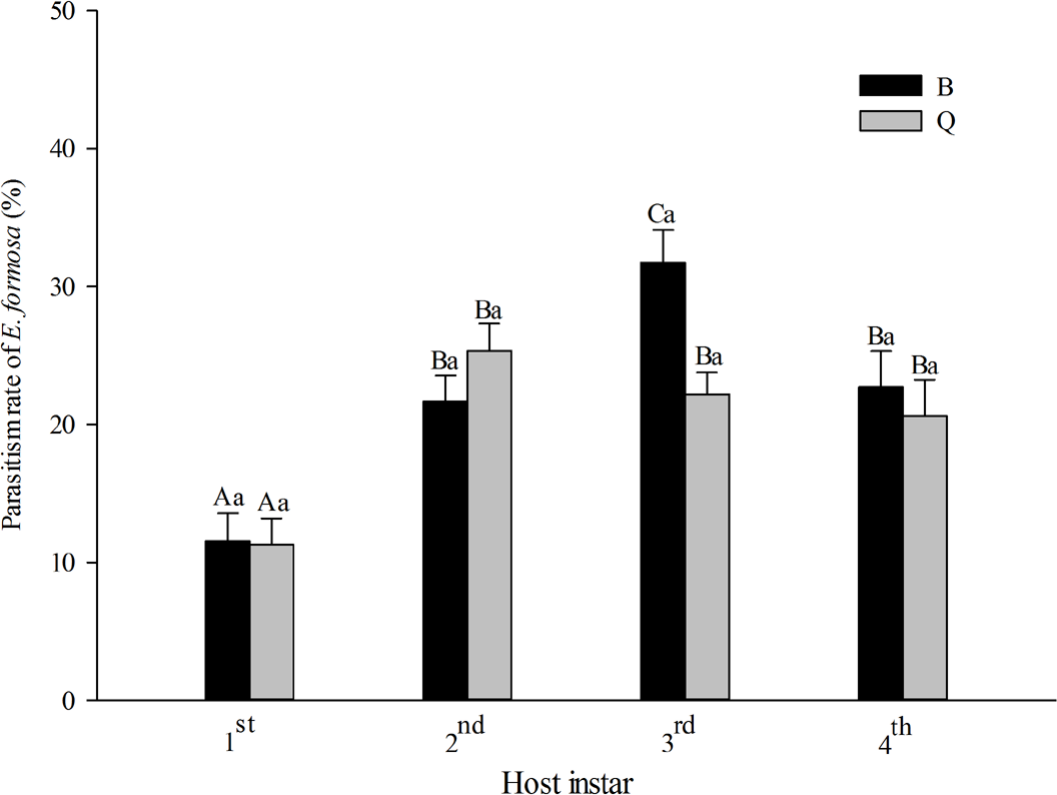
Parasitism rate of *Encarsia formosa* as affected by host biotype (*Bemisia tabaci* biotype Q or B) and instar (1st, 2nd, 3rd, or 4th). Values are means ± SE. Means with different uppercase letters are significantly different within each biotype. Means with different lowercase letters are significantly different within each instar. Tukey test, *p* < 0.05.

The parasitism rate of *E. formosa* was significantly affected by host instar (*F*_3,151_ = 22.43, *P* < 0.0001) but not by host biotype (*F*_1,151_ = 1.65, *P* = 0.200). The interaction between host biotype and host instar was not significant (*F*_3,151_ = 2.50, *P* = 0.061). *Encarsia formosa* parasitized all instars of biotypes B and Q. For biotype B, the parasitism rate was highest on the 3rd instar (31.74%), intermediate on the 2nd instar (21.65%) and 4th instar (22.73%), and lowest on the 1st instar (11.54%). As for biotype Q, the parasitism rate did not significantly differ among the 2nd, 3rd and 4th instars and was significantly lower on the 1st instar (Fig. 1).

### Host-feeding rate of *Encarsia formosa* adults as affected by *Bemisia tabaci* biotype and instar

**Figure 2:**
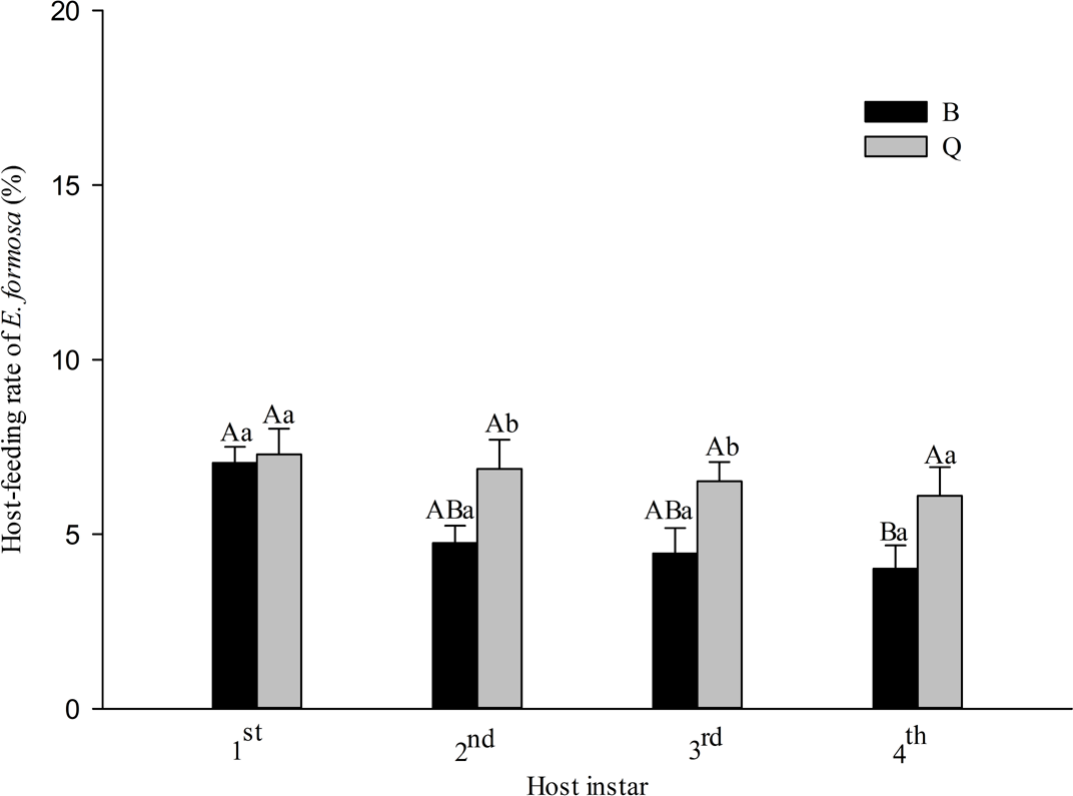
Host-feeding rate of *Encarsia formosa* as affected by host biotype (*Bemisia tabaci* biotype Q or B) and instar (1st, 2nd, 3rd, or 4th). Values are means ± SE. Means with different uppercase letters are significantly different within each biotype. Means with different lowercase letters are significantly different within each instar. Tukey test, *p* < 0.05.

The host-feeding rate of *E. formosa* adults was significantly affected by host biotype (*F*_1,128_ = 12.96, *P* < 0.001) and host instar (*F*_3,128_ = 4.41, *P* = 0.005) but was not significantly affected by the interaction between host biotype and host instar (*F*_3,128_ = 1.69, *P* = 0.173). For biotype B, the host-feeding rate of *E. formosa* adults was highest on the 1st instar (7.04%), intermediate on the 2nd instar (4.75%) and 3rd instar (4.44%), and lowest on the 4th instar (4.01%). For biotype Q, the host-feeding rate did not significantly differ among the four host instars. The host-feeding rate of the parasitoid tended to be higher on all instars of biotype Q than on biotype B, but the difference was significant only for the 2nd and 3rd instars (Fig. 2).

### Immature developmental period of *Encarsia formosa* as affected by *Bemisia tabaci* biotype and instar

**Figure 3:**
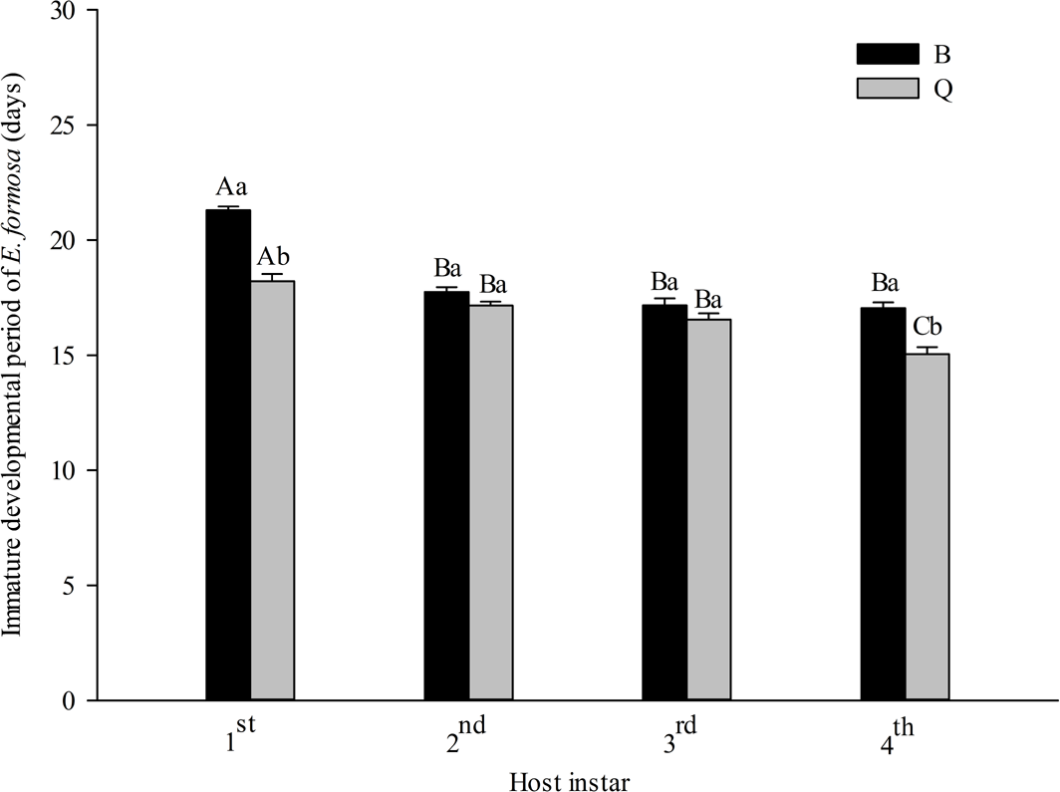
Immature developmental period of *Encarsia formosa* as affected by host biotype (*Bemisia tabaci* biotype Q or B) and instar (1st, 2nd, 3rd, or 4th). Values are means ± SE. Means with different uppercase letters are significantly different within each biotype. Means with different lowercase letters are significantly different within each instar. Tukey test, *p* < 0.05.

The immature developmental period of *E. formosa* was significantly affected by the host biotype (*F*_1,150_ = 77.67, *P* < 0.0001) and host instar (*F*_3,150_ = 76.78, *P* < 0.0001). There was interaction between host biotype and host instar (*F*_3,150_ = 11.60, *P* < 0.0001). For both biotypes of *B. tabaci*, immature developmental period was longer on 1st instar nymphs than on later instars; the immature developmental period was longer (21.30 days) on the 1st instar nymphs of biotype B than on the 1st instar nymphs of biotype Q (18.22 days). The shortest immature developmental period of *E. formosa* occurred on the 4th instar of *B. tabaci* biotype Q. Among the four host instars, the immature developmental period ranged from 17.05 to 21.30 d for biotype B and from 15.05 to 18.22 d for biotype Q, with a downward trend from the 1st to the 4st instar (Fig. 3).

### Emergence rate of *Encarsia formosa* as affected by *Bemisia tabaci* biotype and instar

**Figure 4:**
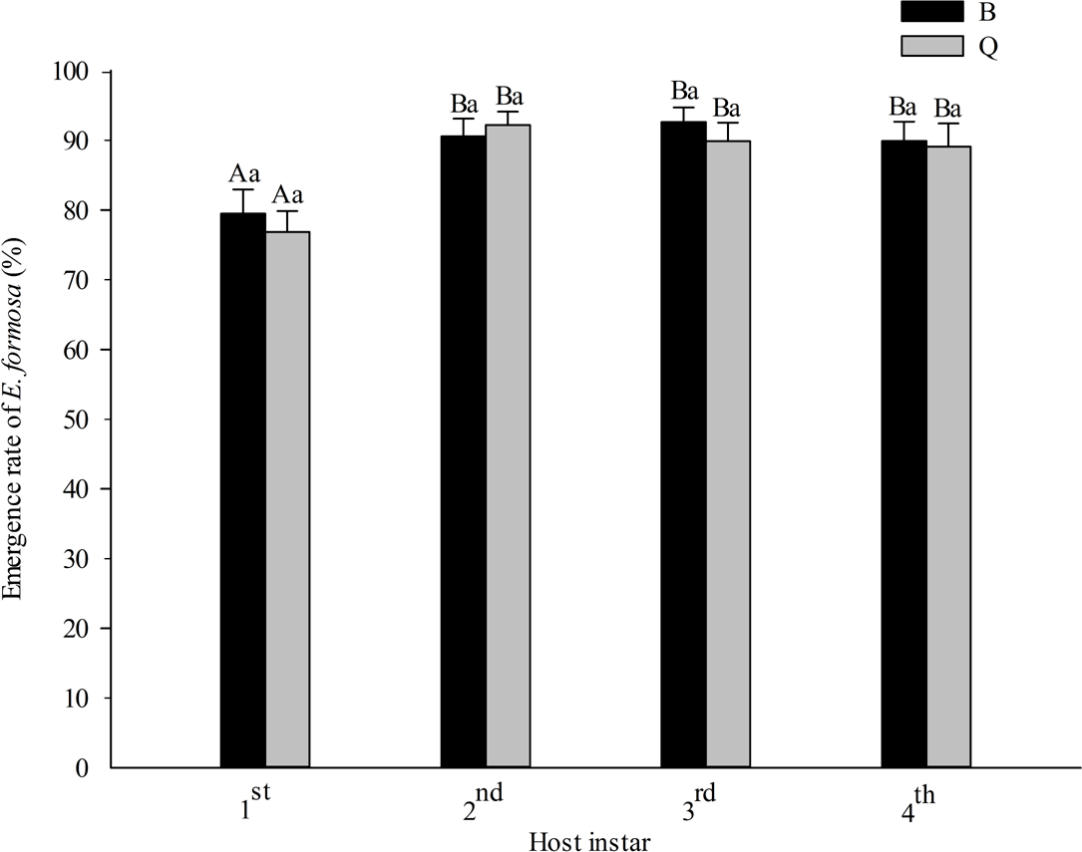
Emergence rate of *Encarsia formosa* as affected by host biotype (*Bemisia tabaci* biotype Q or B) and instar (1st, 2nd, 3rd, or 4th). Values are means ± SE. Means with different uppercase letters are significantly different within each biotype. Means with different lowercase letters are significantly different within each instar. Tukey test, *p* < 0.05.

The emergence rate of *E. formosa* was significantly affected by the host instar (*F*_3,135_ = 10.33, *P* < 0.0001) but not by the host biotype (*F*_1,135_ = 0.25, *P* = 0.616) or by the interaction between biotype and instar (*F*_3,135_ = 0.19, *P* = 0.906). Most *E. formosa* individuals were able to develop to the adult stage regardless of which instar of biotypes Q or B was parasitized. Emergence of *E. formosa* was lower on 1st instars of *B. tabaci* of both biotypes than on later instars. Parasitoid emergence did not differ among the 2nd, 3rd, and 4th instars of *B. tabaci* of either biotype (Fig. 4).

### Longevity of *Encarsia formosa* as affected by *Bemisia tabaci* biotype and instar

**Figure 5:**
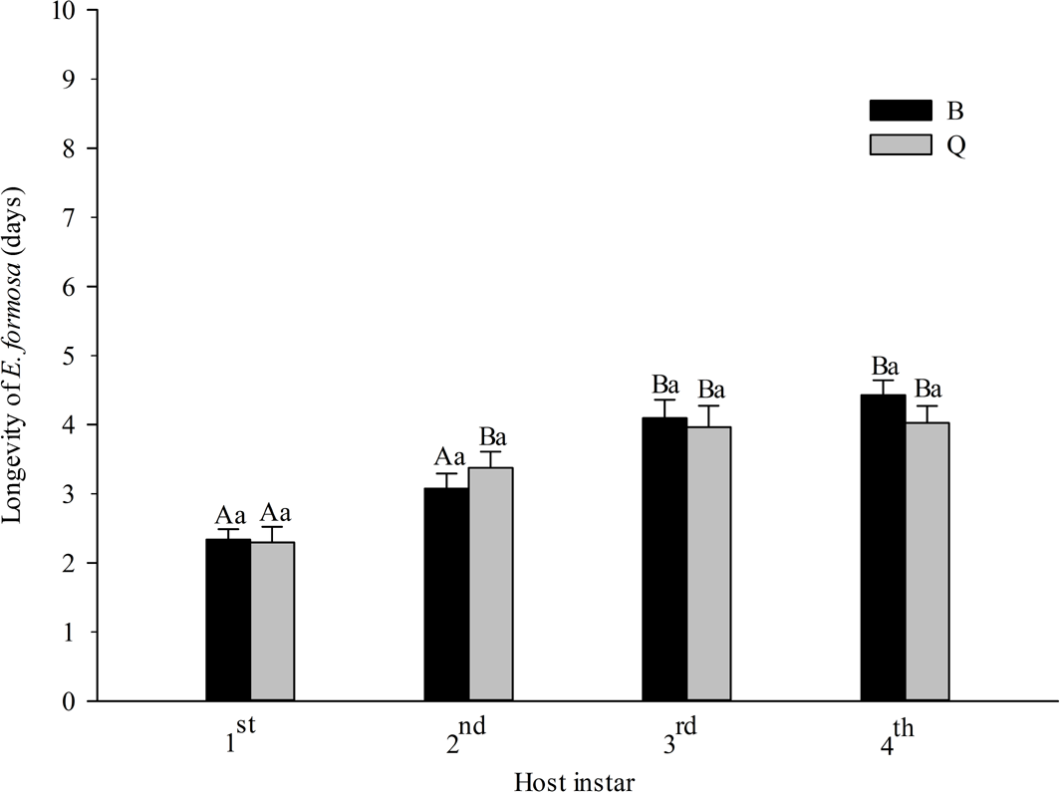
Longevity of *Encarsia formosa* as affected by host biotype (*Bemisia tabaci* biotype Q or B) and instar (1st, 2nd, 3rd, or 4th). Values are means ± SE. Means with different uppercase letters are significantly different within each biotype. Means with different lowercase letters are significantly different within each instar. Tukey test, *p* < 0.05.

The longevity of *E. formosa* was significantly affected by host instar (*F*_3,150_ = 26.67, *P* < 0.0001) but not by host biotype (*F*_1,150_ = 0.17, *P* = 0.684) or the interaction between instar and biotype (*F*_3,150_ = 0.73, *P* = 0.539). On both biotypes, longevity tended to increase with host instar. Parasitoid longevity did not significantly differ between biotype B and Q (Fig. 5).

## Discussion

For parasitoids, differences in the host suitability in terms of initial parasitism, host-feeding, or parasitoid development may result from differences in host quality, which may vary with host age (Colinet et al., 2005). Several models indicated that parasitoid fitness was related to host quality as affected by host size or age (Nicol & Mackauer, 1999; Chau & Mackauer, 2001). The results of the present study indicate that commercially produced *E. formosa* that was mass reared on *T. vaporariorum* could parasitize, feed on, and complete its life cycle on all nymphal stages of *B. tabaci* biotypes B and Q. *Encarsia formosa* preferentially parasitized the 3rd instar of biotype B and the 2nd, 3rd, and 4th instars of biotype Q. Similar results were reported in other studies. *Encarsia formosa* reared on *T. vaporariorum* preferentially parasitized the 3rd instar of B. *argentifolii* (= biotype B) (Qiu et al., 2004). Zhang et al. (2003) reported that two strains of *E. formosa* reared on *B. tabaci* biotype B preferred to parasitize the 3rd instar rather than the other three instars, and Liu et al. (2014) found that the parasitism of *E. formosa* maintained on *B. tabaci* biotype B did not significantly differ between the 2nd and the 3rd instar of *B. tabaci* biotype Q.

A previous report indicated the *E. formosa* reared on *T. vaporariorum* preferred to parasitize the 3rd and 4th instars rather than the 1st and 2nd instars of *B. tabaci* biotype B (Hu, Gelman & Blackburn, 2003). The results of the current study suggest that the rate of parasitism of *E. formosa* was significantly higher on the 3rd instar than on the 4th instar of *B. tabaci* biotype B. In our research, the 4th instar hosts had developed into the 4th instar for 1–2 days before they were used in the experiments. Because *B. tabaci* biotype B and Q do not develop uniformly on tomato plants (Zhang et al., 2014), some of the 4th instar individuals used in our experiments may have developed into pharate adults and thus escaped parasitism, which would cause us to underestimate the rate of oviposition on the 4th instar of *B. tabaci* biotypes B and Q. The rate of parasitism of *E. formosa* was the lowest when the 1st instar of either species was the host, and this result agrees with previous reports (Hu, Gelman & Blackburn, 2003; Zhang et al., 2003). The parasitism rate of the commercially produced *E. formosa* was slightly higher on *B. tabaci* biotype B than on biotype Q, indicating that parasitism by *E. formosa* differs to some extent between the two *B. tabaci* biotypes. We suspect that this may be explained by differences in the nutritional quality or body size between the two whitefly biotypes (X Liu, 2015, unpublished data).

Although it is well known that *E. formosa* not only efficiently parasitizes its hosts but also destructively feeds on them, only a few studies have assessed the feeding rate of *E. formosa* on different *B. tabaci* instars (Liu et al., 2014). The present study found that *E. formosa* mass reared on *T. vaporariorum* had a higher host-feeding rate on 1st instar nymphs than on later instars of *B. tabaci*, which agrees with Wang et al. (2015). The host-feeding rate of *E. formosa* reared on *T. vaporariorum* was higher on biotype Q than on biotype B. This might be related to the time required for a parasitoid to feed on a host and to the difference in body size of biotype B and Q; parasitoids require less time to feed on younger hosts than older hosts probably because the former are smaller (Yang & Wan, 2011; Zhang et al., 2014). Because the parasitoid must consume a greater number of younger hosts than older hosts to obtain enough nutrition to ensure its development, the feeding rate is higher on younger than on older nymphs (Zang & Liu, 2008; Yang & Wan, 2011; Wang et al., 2015). All four instars of *B. tabaci* biotype B are larger than the corresponding instars of biotype Q (unpublished data). This is likely the main reason why *E. formosa* fed on more nymphs of biotype Q than biotype B.

As a koinobiont parasitoid wasp (Nechols & Tauber, 1977; Blackburn, Gelman & Hu, 2002; Hu, Gelman & Blackburn, 2002), *E. formosa* requires a longer time to develop to the adult stage when parasitizing younger rather than older instars (Wang et al., 2015). The immature developmental period (time from oviposition through adult emergence) of commercially produced *E. formosa* decreased as the age of the host instar attacked increased, regardless the biotype (i.e., the immature developmental of *E. formosa* was longer on 1st instars than on later instars). Similar results were reported when *B. tabaci* biotype B and *T. vaporariorum* were parasitized by *E. formosa* reared on *T. vaporariorum* (Hu, Gelman & Blackburn, 2002; Hu, Gelman & Blackburn, 2003; Wang et al., 2015) and when *B. tabaci* biotype B was parasitized by two *E. formosa* strains reared on *B. tabaci* biotype B (Zhang et al., 2003). Differences in *E. formosa* developmental period on different instars of *B. tabaci* biotype B and Q could be due to differences in host size and host developmental rate (Hu, Gelman & Blackburn, 2003), although the environment was constant in the current study.

Most *E. formosa* individuals were able to develop to the adult stage regardless of which instar of biotypes Q or B they parasitized, but the 1st instars of *B. tabaci* of both biotypes were less suitable than the later instars for emergence of *E. formosa*. This result agrees with Hu, Gelman & Blackburn (2003) and Zhang et al. (2003), who found that *E. formosa* emerged more synchronously and at a higher rate when older instars rather than younger instars served as hosts. In other studies, however, the emergence rate of several parasitoids did not differ among host instars (Urbaneja & Stansly, 2004; Liu, 2007; Yang & Wan, 2011). This apparent discrepancy may be due to the differences among parasitoids, host species, experimental methods, host plants, or environmental conditions.

The longevity of *E. formosa* offspring was significantly greater when the 3rd and 4th instars rather than the 1st or 2nd instars of *B. tabaci* biotype B and Q served as hosts, and longevity was shortest with the 1st instar of both whitefly species. These results agree with some previous reports (Hu, Gelman & Blackburn, 2003; Zhang et al., 2003). We speculate that the female parasitoids would probably choose host instars (e.g., the 3rd and 4th instars) that contained more nutrition for prolonging the longevity of progenies (Colinet et al., 2005; Luo & Liu, 2011; Wang et al., 2015).

The present study demonstrates that parasitic or host-feeding characteristics of *E. formosa* that was commercially produced using *T. vaporarioum* differed between the biotypes B and Q of *B. tabaci*. Other reports involving several host-parasitoid interactions indicate that the host species used to rear parasitoids affects its subsequent preference for hosts (Luo & Liu, 2011; Wang et al., 2013; Dai et al., 2014; Wang et al., 2015). In a system involving *Phaseolus vulgaris* L. (Ambra’), the whitefly *B. tabaci*, and the parasitoid *Encarsia sophia* Girault and Dodd (Hymenoptera: Aphelinidae), almost all biological parameters of *E. sophia* were superior when the parasitoid was maintained on *T. vaporarioum* rather than on *B. tabaci* (Luo & Liu, 2011). *Eremocerus hayati* Zolnerowich and Rose maintained on *B. tabaci* biotype B preferred to feed on the 1st, 2nd, and 3rd instars of *B. tabaci* biotype Q rather than the 4th instar nymph, and preferred to parasitize the 2nd instar of *B. tabaci* biotype Q rather than the other instars (Zhang et al., 2014). *Eremocerus hayati* reared on *B. tabaci* biotype Q had a significantly higher host-feeding rate on the 1st instar of *B. tabaci* biotype Q than on the other three instars, and preferred to parasitize 2nd and 3rd instars of biotype Q rather than the 1st and 4th instars (Dai et al., 2012). In contrast, the clutch size, sex ratio, and fitness of the first generation of *Hyssopus pallidus* Askew (Hymenoptera: Eulophidae), a gregarious ectoparasitoid of the tortricid moths *Cydia molesta* Busck and *Cydia pomonella* L. (Lepidoptera, Tortricidae), were not influenced by original rearing host species even though those species differed considerably in size (Johanna, Rott & Silvia, 2007).

In conclusion, this study showed that parasitism of *B. tabaci* by *E. formosa* commercially produced on *T. vaporariorum* differed in some respects depending on whitefly biotype (B or Q) and host instar. Our results indicate that the commercially-produced *E. formosa* is able to control all instars of *B. tabaci* biotypes B and Q, making this parasitoid a promising tool for the management of the two biotypes in China. Where *B. tabaci* biotype B and Q coexist in the field (Chu et al., 2010; Pan et al., 2011) and whether commercially-produced *E. formosa* preferentially parasitizes one biotype or the other remains to be determined.

## Supplemental Information

10.7717/peerj.1863/supp-1 Data S1 Raw data

## Additional Information and Declarations

### Data Availability

The following information was supplied regarding data availability: Raw data has been uploaded as Supplemental Information.
